# Choosing the Right Biologic for the Right Patient With Severe Asthma

**DOI:** 10.1016/j.chest.2024.08.045

**Published:** 2024-09-06

**Authors:** Simon Couillard, David J. Jackson, Ian D. Pavord, Michael E. Wechsler

**Affiliations:** a Faculté de Médecine et des Sciences de la Santé, Université de Sherbrooke, Sherbrooke, QC, Canada; b Guy’s Severe Asthma Centre, Guy’s and St Thomas’ Hospitals, London, England; c School of Immunology and Microbial Sciences, King’s College, London, England; d Respiratory Medicine Unit and Oxford Respiratory NIHR BRC, Nuffield Department of Medicine, University of Oxford, Oxford, England; e National Jewish Health, Denver, CO

**Keywords:** adherence, airways, asthma, biologics, biomarkers, eosinophils, exacerbations, Feno, multidisciplinary team, severe asthma, type 2 inflammation

## Abstract

In this installment of the How I Do It series on severe asthma, we tackle the clinical conundrum of choosing the right biologic for the right patient with severe asthma. With six biologics now approved for use in this area comprising four different targeting strategies (anti-Ig E: omalizumab; anti-IL-5 and anti-IL-5-receptor: mepolizumab, reslizumab, and benralizumab; anti-IL-4-receptor: dupilumab; anti-thymic stromal lymphopoietin: tezepelumab), this question is increasingly complex. Recognizing that no head-to-head trial has compared biologics, we based our review on the expected effects of inhibiting different aspects of type 2 airway inflammation, supported whenever possible by clinical trial and real-world data. We use four variations of a case of severe uncontrolled asthma to develop concepts and considerations introduced in the previous installment (“Workup of Severe Asthma”) and discuss pregnancy-related, biomarker-related, comorbidity-related, and corticosteroid dependency-related considerations when choosing a biologic. The related questions of deciding when, why, and how to switch from one biologic to another also are discussed. Overall, we consider that the choice of biologics should be based on the available clinical trial data for the desired efficacy outcomes, the biomarker profile of the patient, safety profiles (eg, when pregnancy is considered), and opportunities to target two comorbidities with one biologic. Using systemic and airway biomarkers (blood eosinophils and exhaled nitric oxide [Feno]) and other phenotypic characteristics, we suggest a framework to facilitate therapeutic decision-making. Post hoc studies and new comparative studies are needed urgently to test this framework and to determine whether it allows us to make other clinically useful predictions.

Until the early 2010s, the asthmatologist’s role in the severe asthma clinic was largely that of a "corticosteroid-ologist." In this capacity, we studied the inevitable airway and people remodelling in chronically debilitated patients with asthma, with the uncomfortable suspicion that a portion of the morbidity was the result of corticosteroids—substances known for both their efficacy and toxicity.^1^, ^2^, ^3^ Thankfully, the advent of multiple different monoclonal antibodies to treat people with asthma has translated to extraordinary benefits for patients, and a less uncomfortable workday for the asthmatologist. An important aspect of today’s job involves phenotyping patients clinically (by looking for patient characteristics and comorbidities) and endotyping objectively (by measuring type 2 inflammatory biomarkers) to decipher the underlying mechanisms of their airway disease, estimating the risk of asthma attacks and lung function decline, and most importantly, offering a precision-based approach by choosing the right treatment for the right patient.^4^ As the variety of biologic therapies increases and now includes six biologics spanning four different mechanisms of action, the latter task is increasingly complicated.

In a previous instalment of the severe asthma How I Do It series about the workup of severe asthma,^5^ we proposed a "two-hit model" by which blood eosinophils and exhaled nitric oxide (Feno) identify different components and compartments of the inflammatory response. We elaborated on the increasing volume of evidence that allergy testing and serum IgE have poor prognostic and theragnostic value in asthma; we reviewed the complementary value of type-2 inflammatory biomarkers (blood eosinophils and Feno), spirometry, and adherence checking, and we emphasized the importance of evaluating and managing treatable type 2 comorbidities.

In this installment, we tackle how different phenotypic characteristics can help us to find the right biologic for the right patient. In complement to recent comprehensive^6^ and useful^7^ reviews on the currently authorized biologics that we list in Table 1, we use case studies highlighting four different sets of clinical scenarios and biomarker values to discuss features influencing our choice of targeted therapy.

**Table 1:** Therapeutic Strategies of Biologics Approved in Asthma, Biomarkers for Selection Criteria, and Clinical Efficacy Outcomes

Therapeutic Strategy (Biologic)	Selection Criteria	Asthma Attacks	OCS Sparing	ACQ	FEV_1_	AHR	Comorbidities	Comments
Anti-IgE (omalizumab)	Serum IgE 30-700 with perennial aeroallergen sensitization	+	Unclear	+	0	0	CSU ++ CRSwNP + Food allergy ++	Mostly studied in moderate asthma, less in very severe
Anti-IL-5 and anti-IL-5R (mepolizumab, reslizumab, benralizumab)	Blood eosinophils ≥ 0.15 ×10^9^ cells/L at screening or ≥ 0.3 × 10^9^/L in past year	++	++	+	+	0/+	EGPA^a^ ++ HES^a^ ++ CRSwNP +	Major clinical effects proportional to eosinophilia
Anti-IL-4 and anti-IL-13 (dupilumab)	Blood eosinophils ≥ 0.15 × 10^9^/L, Feno ≥ 25 ppb, or both	++	++	+	++	Unclear^b^	AD ++ CRsNP ++ EoE ++ PN ++	Induces transient eosinophilia independent of efficacy
Anti-TSLP (tezepelumab)	None^c^	++	0	+	++	+	?	Major efficacy when blood eosinophils, Feno, or both are raised; lesser yet significant efficacy when biomarkers low

ACQ = Asthma Control Questionnaire; AD = atopic dermatitis; AHR = airway hyperresponsiveness; CRSwNP = chronic rhinosinusitis with nasal polyposis; CSU = chronic spontaneous urticaria; EGPA = eosinophilic granulomatosis with polyangiitis; EoE = eosinophilic esophagitis; Feno = fraction of exhaled nitric oxide; HES = hypereosinophilic syndrome; ICS = inhaled corticosteroid; OCS = oral corticosteroid; PN = prurigo nodularis; TSLP = thymic stromal lymphopoietin; + = clinically improved; 0 = measured and minimal effect observed; ? = not measured.

a Mepolizumab is authorized for use in EGPA and HES; benralizumab has shown positive results in a phase 3 trial in EGPA^8^(label pending) and is authorized for use in HES.

b Measured, not enough data points for conclusion.

c Asthma attacks defined as acute asthma requiring ≥ 3 days of systemic corticosteroid treatment.

## Master Case Overview

Please evaluate this patient with asthma and three severe asthma attacks in the prior year despite taking a combination of high-dose inhaled corticosteroid (ICS), long-acting β_2_-agonist, and long-acting antimuscarinic. In clinic, the Asthma Control Test Questionnaire score is 14, FEV_1_ after bronchodilator administration is 65% predicted with 15%/352 mL reversibility, and FEV_1_ to FVC ratio of 0.57. Other data, including values of type 2 inflammatory biomarkers, are listed below.

### Case Report Variation 1

This patient is a 35-year-old woman with childhood-onset asthma and the following biomarker profile: blood eosinophil count, 0.35 × 10^9^/L; Feno, 45 parts per billion (ppb); serum IgE, 350 kU/L; with positive skin test results for house dust mite.

#### Case Report 1 Discussion

##### Workup

In line with the first part of this severe asthma article series,^5^ many elements are given that confirm the clinical problem: uncontrolled type 2 high-inflammation asthma with evidence of allergic sensitization. We emphasize that atopy is a clinical definition based on allergic symptoms plus sensitization. The two missing workup elements are a confirmation of good adherence and a review of imaging. An assessment of inhaler technique and ICS adherence is critical to avoid unnecessary escalation of treatment to costly biologic therapies and can be assessed most easily by counting prescription refills. Adherence ideally is checked before meeting the patient, which allows for a prompt and frank conversation about nonadherence when identified. However, because a prescription does not necessarily equate to daily ICS use, if concern exists that poor control simply may reflect poor ICS adherence (as opposed to genuine severe asthma), a more robust objective evaluation such as ICS prescription refills, connection to an inhaler e-monitor,^9^ or an Feno suppression test^10^ is cost-effective.^11^^,^^12^ In general, Feno of ≥ 40 ppb or a ≥ 20% increase in its value should prompt a review of adherence and inhaler technique.^10^^,^^13^ An up-to-date chest radiograph should suffice for imaging in view of her young age and the straightforward presentation of the disease. A high-resolution CT scan would be indicated if abnormalities appeared on the chest radiograph or atypical features were included in the history, such as a high exacerbation rate without elevated T2 biomarkers, a pulmonary function test pattern not fully consistent with obstruction, an absence of prednisolone-responsive symptoms, or a combination thereof.

##### Making the Decision for a Biologic

Assuming a satisfactory and unrevealing workup, four main therapeutic options could be considered at the first patient encounter. In increasing order of expected benefits for the patient, these are as follows.

1. No therapeutic change is implemented, but allergen avoidance is recommended, despite the low likelihood that that will decrease exacerbation frequency.^14^ Ordering more ancillary tests may be considered, despite having clearly identified the clinical problem.^5^
2. Optimizing the pharmaceutical care of potentially related comorbidities (eg, acid reflux, sinus disease), without necessarily addressing the cause of asthma attacks.^15^
3. Prescribing oral medications (eg, montelukast, sublingual immunotherapy,^16^ or theophylline^17^) that are likely to be of very limited proven efficacy in severe asthma.
4. Initiating a monoclonal antibody for asthma. Although all of the options above may be considered, this is likely the best option, assuming an appropriate prescription based on biomarkers and comorbidities and that the patient meets regional prescription criteria, treatment is reimbursed, and the medications are available.

In considering biologic therapies in this patient and in all ensuing patients, we assume that the extrinsic factors noted in (4) otherwise have been addressed.

##### Choosing a Biologic

The first patient is typical insofar as she is eligible for all biologics available: the anti-IgE omalizumab, the anti-IL-5s mepolizumab and reslizumab, the anti-IL-5-receptor (IL-5R) benralizumab, the anti-IL-4-receptor dupilumab, and the anti-thymic stromal lymphopoietin (TSLP) tezepelumab.

In the absence of direct head-to-head analyses, it is difficult to provide a strong evidence-based choice solely considering the characteristics of the asthma. As can be expected, indirect comparisons of different biologic treatments either by carefully matching key baseline characteristics or by performing network meta-analyses have not yet led to any firm conclusions.^18^, ^19^, ^20^ Perhaps the sole exception to this rule is when a treatment goal is to wean patients off maintenance systemic corticosteroids; mepolizumab, benralizumab, and dupilumab have demonstrated oral corticosteroid (OCS)-sparing effects in controlled trials.^21^, ^22^, ^23^ However, this is not applicable to this patient’s case. So, how does one choose?

First, consider the biomarkers: both circulating eosinophil counts and Feno are raised, identifying two immunologic pathways that could be implicated in the pathophysiologic characteristics,^5^^,^^24^ and thus identifying a high-risk and high-stake situation for the patient. The high risk relates to the predictable potential for future asthma attacks, whereas the stakes are high because the same type 2 inflammatory process that predicts an excellent response to type-targeted antiinflammatory medication also puts the patient in harm’s way.^25^, ^26^, ^27^, ^28^, ^29^ Dupilumab, tezepelumab, and, to a lesser extent, omalizumab can influence both eosinophils and Feno,^30^, ^31^, ^32^, ^33^ whereas anti-IL-5 and anti-IL-5R will not suppress Feno.^34^^,^^35^ The recent Study to Assess the Reduction of Daily Maintenance ICS/LABA Treatment Towards Anti-Inflammatory Reliever Treatment in Patients With Severe Eosinophilic Asthma Treated With Benralizumab (SHAMAL) trial, in which weaning of ICS while taking benralizumab was associated with an Feno-related decrease in FEV_1_, suggests this might be an important factor.^34^

One also should take into account the early onset of asthma in this patient. With the results of the Evaluation of Dupilumab in Children With Uncontrolled Asthma (VOYAGE)^36^ and Trial of Mepolizumab Adjunctive Therapy for the Prevention of Asthma Exacerbations in Urban Children (MUPPITS2)^37^^,^^38^ studies in children, in addition to post hoc analyses of phase 3 trials of benralizumab,^39^ the early onset of the disease and raised Feno level may imply that the patient has epithelial or atopic-driven type 2 inflammation. This phonotype suggests that she would benefit more from Feno and IgE-lowering therapies (eg, inhibition of IL-4 or IL-13 by dupilumab or, more speculatively, binding of TSLP with tezepelumab)^36^ than blood eosinophil-lowering therapies (eg, inhibition of IL-5 or IL-5R by mepolizumab or benralizumab).^37^, ^38^, ^39^ Indeed, Feno increases with IL-13 activity in the airway epithelium^40^—most classically with allergies, the hallmark of childhood asthma^41^—whereas the blood eosinophil count reflects systemic IL-5 activity and the pool of effector cells,^24^ a key biomarker for severe adult-onset asthma.^41^^,^^42^ It would be interesting to ask the patient about her response to prednisolone given to treat attacks. A prompt and complete response confirms that type 2 inflammation is playing an important role and augurs well for a response to type 2-targeting biologics.^43^

Arguably, the key to this case lies in the first four words of the case description: (1) a woman of child-bearing age, urging safety and teratogenic considerations, with (2) early-onset asthma, suggesting epithelial or atopic-drive disease. If she is actively considering pregnancy, it seems reasonable to base our choice of biologic first on teratogenicity safety profiles.

##### Pregnancy-Related Considerations

Prescribers can be reassured that no concerns of teratogenicity for biologics have been substantiated, despite the drugs undergoing trials and being used in various diseases for > 2 decades.^44^ Nevertheless, all these treatments are IgG based, and thus are transported across the placenta in varying degrees according to gestational age and subtype.^45^^,^^46^ Thus, we would rather choose a biologic with the most robust safety profile.

Omalizumab has been administered to humans since 1995^47^ and has been marketed since 2003.^48^ It has a long history of safety in pregnancy. An analysis of a prospective exposure cohort registry of 250 pregnant women with asthma treated with this biologic showed no increase in adverse fetal outcomes when compared with a disease-matched external cohort.^49^ Although the magnitude of exacerbation reduction and corticosteroid sparing may be lower,^50^ omalizumab is a safer choice for women hoping to become pregnant.^44^^,^^51^ In the setting of an early-onset atopic phenotype with no steroid dependency such as in patient 1, it is a reasonable choice.

IL-5*-* and IL*-*5R*-*targeting agents include mepolizumab, reslizumab, and benralizumab*.* Mepolizumab has undergone trials in humans since 2000.^52^ Its growing clinical experience, presence in registries, and the publications in which it is a topic are very reassuring. No signal of harm in pregnancy and breastfeeding has been published. In pregnant nonhuman primates, administration of mepolizumab and benralizumab surrogate-antibody doses of ninefold and 310-fold of the maximum recommended human dose elicited no maternal or fetal adverse effect up to 9 months after birth.^53^^,^^54^ No such data are available for reslizumab, whose administration is IV and thus less practical.^55^ Although mepolizumab or benralizumab are adequate choices for this patient, they would be our first choice only if she were corticosteroid-dependent (Table 2).^21^, ^22^, ^23^^,^^56^

**Table 2:** Summary of Corticosteroid-Sparing Effects of Biologics Based on Randomized Controlled Trials

Biologic	Trial	Median Reduction in OCS Dose With Active and Placebo, %	Change in FEV_1_ Before Bronchodilator Administration Active Minus Placebo, mL	Reduction in Asthma Attack Rates, %
Omalizumab	NA	NA	NA	NA
Mepolizumab^23^	SIRIUS	50 vs 0	114	32
Benralizumab^22^	ZONDA	75 vs 25	113	55
Dupilumab^1^	VENTURE	100 vs 50	220	59
Tezepelumab^56^	SOURCE	Not significant (100 vs 75)	260	Not significant (31)

Data from references 21-23, and 56. NA = not available; OCS = oral corticosteroid; SIRIUS = Oral Glucocorticoid-Sparing Effect of Mepolizumab in Eosinophilic Asthma; SOURCE = Study to Evaluate the Efficacy and Safety of Tezepelumab in Reducing Oral Corticosteroid Use in Adults With Oral Corticosteroid Dependent Asthma; VENTURE = Efficacy and Safety of Dupilumab in Glucocorticoid-Dependent Severe Asthma; ZONDA = A Multicenter, Randomized, Double-Blind, Parallel Group, Placebo-controlled, Phase 3 Efficacy and Safety Study of Benralizumab (MEDI-563) to Reduce Oral Corticosteroid Use in Patients With Uncontrolled Asthma on High Dose Inhaled Corticosteroid Plus Long-acting β2 Agonist and Chronic Oral Corticosteroid Therapy.

Dupilumab and tezepelumab inhibit the type 2 inflammatory cascade proximally by binding the IL-4-receptor and TSLP, respectively. These biologics have a broad range of pathophysiologic and clinical effects in multiple immune compartments, two factors that temper their consideration when considering potential pregnancy. Nevertheless, in pregnant nonhuman primates, doses 10 and 168 times the maximum recommended human dose have been administered with no adverse outcome noted.^57^^,^^58^ In women of child-bearing age planning to have children, we prefer to initiate a more pregnancy-established biologic, as noted. If an unplanned pregnancy occurs while a patient is taking these therapies, an informed decision taken with the patient and the multidisciplinary team, pharmacovigilance, spontaneous reporting of suspected adverse drug reactions, and discussing registry enrolment are crucial.^59^

#### Case Report 1 Conclusions

**Figure 1:**
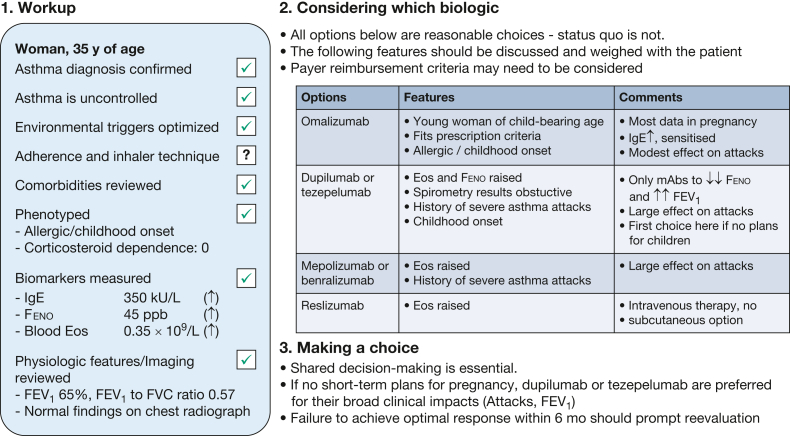
Framework for choosing a biologic, applied to the patient from case study variation 1. The workup of this patient needs to be completed by an adherence check and a review of imaging. She has severe uncontrolled allergic asthma, and with her biomarker profile, she is in a high-risk and high-stake situation, but with no noteworthy comorbidity (Table 1). She is eligible and would respond to all marketed mABs. A key element to guide our therapeutic choice is the potential for pregnancy in a young woman of child-bearing age. If she has short-term or medium-term plans for pregnancy, we recommend omalizumab; if not, we prefer dupilumab or tezepelumab. Eos = eosinophil count; Feno = fractional exhaled nitric oxide; mAB = monoclonal antibody.

Because multiple biologics are indicated in this patient, shared decision-making is essential (Fig 1). For example, the injection schedule (every 2, 4, 8, or 26 weeks)^60^ and other logistical aspects (reimbursement, speed of initiation) may carry significant weight in the decision process. In this woman of childbearing age with uncontrolled allergic type 2 high-inflammation asthma, our choice largely would hinge on her plans for pregnancy. If she has no such plans in the short term, we certainly recommend an anti-IL-4 or anti-TSLP (an anti-IL-4-receptor would be preferable if she were corticosteroid dependent). If pregnancy is possible in the short term, the anti-IgE omalizumab is the safest option, although this will have lesser clinical impact on exacerbations and FEV_1_. Safety of a biologic in pregnancy is important, and we recognize that many clinicians may not feel comfortable initiating these drugs in women planning to become pregnant. Nevertheless, it is equally important to balance asthma control and to minimize exacerbations and OCS sparing, which can have an adverse impact on viability and pregnancy outcomes.^44^ Thus, it would be most prudent to start a biologic here. Exceptionally refractory cases in young women hoping to become pregnant may yet prompt consideration for dupilumab or tezepelumab to avoid the negative effects of asthma attacks and OCS, despite lack of robust safety data in pregnancy.

### Case Report Variation 2

This patient is a 56-year-old woman with adult-onset asthma and the following biomarker results: blood eosinophil count, 0.1 × 10^9^/L; Feno, 15 ppb; serum IgE, 18 kU/L; and negative skin test results for common aeroallergens.

### Case Report 2 Discussion

This patient has confirmed nonallergic, noneosinophilic type 2 low-inflammation (ie, non-type 2) asthma with high-dose ICS, and we presume that adequate adherence has been confirmed. This form of asthma occurs in 5% to 40% of patients with severe asthma,^61^ although real-world data^62^ and a trial showing that biomarker-directed withdrawal of antiinflammatory therapy unmasks a type 2 phenotype in 95% of patients with truly severe asthma,^63^ suggest that true type 2 low-inflammation severe asthma is quite uncommon.

Although this biomarker profile usually is associated with a low risk of severe exacerbations or lung function decline in the placebo arms of trials in clinical trials in severe asthma,^25^^,^^26^^,^^29^^,^^31^^,^^64^^,^^65^ this patient experienced three attacks/y despite a type 2 low-inflammation phenotype. Therefore, it is very important to assess this patient during an asthma attack to assess the cause of the clinical problem objectively.^66^ For example, chronic airways infection predicts an excellent response to thrice weekly azithromycin^67^, ^68^, ^69^ at far lesser cost than a biologic, although the absence of mycobacteria should be confirmed first to avoid inducing macrolide resistance. Even in the absence of positive sputum culture findings, azithromycin is effective in preventing asthma attacks.^69^ At other times, the recurrent airway crises may reflect "pseudoasthma" or a combination of asthma and other entities, such as severe gastroesophageal acid reflux disease, aspiration, anxiety, dysfunctional breathing, or inducible laryngeal obstruction.^5^ If, however, variable airflow limitation at the time of the asthma attack is indeed present in this person with type 2 low-inflammation disease, then the question of which biologic to choose remains.

**Figure 2:**
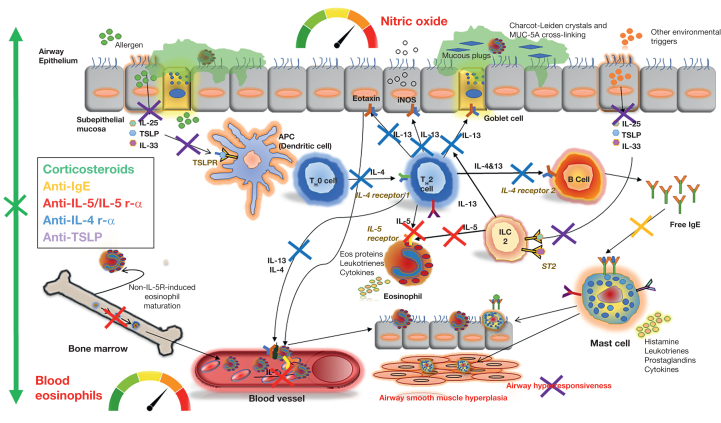
Diagram showing type 2 inflammatory cascade in severe asthma and the effects of antiinflammatory therapies. The type 2 immune response may be set off by a trigger (eg, allergen, smoke or pollution, infection) in the airways, leading to epithelial alarmin signalling with downstream type 2 cytokine (IL-5, IL-4, and IL-13) activity and migration of circulating eosinophils to the airways in most patients, and non-type 2 mechanisms in others (eg, mastocyte activation). In type 2 inflammatory severe asthma, blood eosinophils reflect circulating IL-5 and the systemic pool of available effector cells, whereas fractional exhaled nitric oxide reflects IL-13 activity in the airway compartment (mucus hypersecretion, bronchial motor tone, and chemotaxis of eosinophils).^5^^,^^24^^,^^34^^,^^64^ Corticosteroids have broad biologic and clinical effects at the cost of associated toxicity. Targeting the end products of the type 2 pathway, such as IgE, has had modest success in asthma. In the past decade, a strategy based on blocking progressively more proximal drivers of inflammation such as IL-5, IL-4, IL-13, and TSLP has proven successful. APC = antigen presenting cell; Eos = eosinophils; MUC-5A = mucin 5-A; iNOS = inducible nitric oxide synthase; TSLP = thymic stromal lymphopoietin; TSLPR = thymic stromal lymphopoietin receptor.

The antialarmin, anti-TSLP tezepelumab is the first biologic to show clinical efficacy across all phenotypes, and thus represents the only evidence-based choice for this patient. Indeed, in the phase 3 randomized controlled Study to Evaluate Tezepelumab in Adults & Adolescents With Severe Uncontrolled Asthma (NAVIGATOR) trial of severe asthmatics with recurrent exacerbations,^31^ 1,061 patients were randomized to tezepelumab or placebo. After 52 weeks, tezepelumab reduced the annualized severe asthma attack rate by 56% compared with placebo in the intention-to-treat population. The reduction was observed predominantly in people with type 2 inflammatory asthma, but remained statistically significant in the subgroup with low type 2 biomarkers. These observations were confirmed in a pooled analysis of NAVIGATOR and the phase 2 trial Study to Evaluate the Efficacy and Safety of MEDI9929 [AMG 157] in Adult Subjects With Inadequately Controlled, Severe Asthma (PATHWAY),^70^ in which participants with high biomarkers (blood eosinophil count ≥ 0.3 × 10^9^/L and Feno ≥ 25 ppb) showed an impressive 77% reduction in asthma attack rate equating to 2.2 fewer attacks per year, whereas the people with low biomarkers (blood eosinophil count < 0.15 × 10^9^/L and Feno < 25 ppb) achieved a modest 35% to 38% relative reduction, equating to 0.4 fewer attacks per year. The difference in relative rate reductions highlights that tezepelumab acts mainly via the inhibition of epithelial-derived alarmin TSLP and subsequent shutdown of downstream type 2 inflammation, an observation supported by signals observed in the translational Study to Evaluate Tezepelumab on Airway Inflammation in Adults With Uncontrolled Asthma (CASCADE)^71^ and Effects of Anti-TSLP in Patients With Asthma **(**UPSTREAM)^72^ study (Fig 2). The substantial difference in absolute rate reductions also is the result of the lower risk of asthma attacks in the patients with type 2 low-inflammation asthma who received placebo compared with those with type 2 high-inflammation disease.^29^^,^^73^

#### Case Report 2 Conclusions

In this middle-aged woman with confirmed nonallergic adult-onset type 2 low-inflammation severe asthma, we first would consider carefully the possibility of airway infection or other comorbidities explaining the recurrent asthma attacks. Hence, we routinely review and phenotype patients with type 2 low-inflammation disease at the time of an acute episode and obtain sputum cultures. As soon as airway infection and comorbidities are excluded, the recommendation is for tezepelumab: the only biologic with some evidence of efficacy in severe asthma patients with low biomarker levels.

### Case Report Variation 3

This patient is a 46-year-old man with adult-onset asthma requiring maintenance prednisolone at a dose of 10 mg once daily for the past 2 years. Biomarker profile reveals a blood eosinophil count of 0.16 × 10^9^/L, Feno of 125 ppb, serum IgE of 180 kU/L, and negative skin test results for common aeroallergens. Historical blood eosinophil counts were raised in the range 0.4 to 0.6 × 10^9^/L before starting regular prednisolone; Feno consistently is raised in the range of 120 to 150 ppb, with no evidence of a response to supervised high-dose ICS treatment and only a limited response to prednisolone. This patient also has evidence of chronic rhinosinusitis with nasal polyposis. Antineutrophil cytoplasmic antibodies findings are negative.

#### Case Report 3 Discussion

This is a typical presentation of severe adult-onset asthma with type 2 inflammation and OCS dependence. Upper airway involvement is common and high blood eosinophil counts and Feno levels invariably are seen. The lower and upper airway disease typically is very responsive to OCSs and less responsive to even optimally delivered topical steroids. In this case, showing a lack of Feno suppression despite high dose ICSs, the airway mucosal processes driving type 2 airway inflammation are resistant to ICS treatment.^74^ Consequently, effective treatment has to exploit other mechanisms to reduce airway inflammation: depletion of the reservoir of circulating eosinophils (oral corticosteroids and biologics targeting the IL-5 pathway: mepolizumab, benralizumab, and reslizumab), prevention of eosinophils leaving the vascular compartment (targeting the IL-4-receptor-alpha and inhibiting IL-4 and IL-13 responses: dupilumab), or inhibition of the corticosteroid-resistant airway mucosal drivers of type 2 inflammation by other mechanisms (targeting TSLP with tezepelumab).^75^ We note a blood eosinophil count of 0.16 × 10^9^/L, likely underestimated because of ongoing OCS exposure. Accordingly, a historical blood eosinophil count of ≥ 1.5 × 10^9^/L favors an anti-IL-5 or IL-5R because of the possibility of hypereosinophilic syndrome or eosinophilic granulomatosis with polyangiitis and contraindicate the use of dupilumab. As a further discussion point, in people not receiving maintenance OCS, marginal eosinophilia (0.15-≤ 0.30 × 10^9^/L) is associated with poorer response to anti-IL-5 of IL-5R^76^^,^^77^ compared with upstream-acting agents dupilumab or tezepelumab.^30^^,^^70^

The main goal of treatment is to allow the patient to withdraw from prednisolone treatment without losing control of the asthma. An additional consideration is that the treatment choice also might bring the nasal disease under control. Although omalizumab is approved independently for chronic rhinosinusitis with nasal polyposis, the lack of perennial allergic sensitization and the lack of OCS-sparing effect with omalizumab make this a less suitable choice. Mepolizumab, benralizumab, and dupilumab have proven OCS-sparing effects, with placebo-controlled trials showing rather similar efficacy (Table 2).^21^, ^22^, ^23^ All improve underlying asthma control and lung function, despite a reduction in oral prednisolone dose with numerically greater reductions in exacerbations seen with benralizumab and dupilumab and a numerically superior improvement in lung function seen with dupilumab (Table 2).

#### Case Report 3 Conclusions

**Figure 3:**
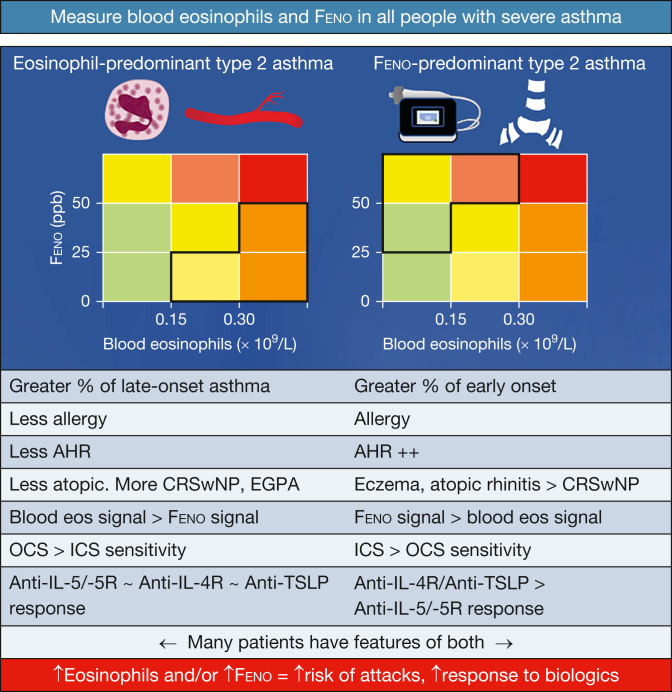
Diagram showing potential features of blood eosinophil and Feno-predominant type 2 high-inflammatory asthma. All patients with severe asthma should have blood eosinophils and Feno evaluated. Both sets of characteristics represent a spectrum, and patients may show features of both. Patients with elevations in either biomarker are at increased risk of exacerbations and are more likely to respond to biologic therapies; combined elevations of both are worse than single-biomarker elevation. In general, patients with raised Feno have earlier onset, allergic asthma characterized by more airway hyperresponsiveness, more allergy and allergy-associated comorbidities, and a more complete response to ICS and to anti-TSLP and anti-IL-4R. The figure content is adapted and speculative based on the data discussed in Couillard et al.^75^ AHR = airway hyperresponsiveness; CRSwNP = chronic rhinosinusitis and nasal polyposis; EGPA = eosinophilic granulomatosis and polyangiitis; eos = eosinophils; Feno = fractional exhaled nitric oxide; ICS = inhaled corticosteroid; IL-4R = IL-4 receptor; OCS = oral corticosteroid; ppb = parts per billion; TSLP = thymic stromal lymphopoietin.

This case report demonstrates how comorbidities often drive the choice of the biologic. Indeed, mepolizumab or benralizumab would be reasonable choices if we consider that the patient’s blood eosinophil count is very likely to be high again when the oral prednisolone dose is lowered. However, the absence of historical hypereosinophilia, the finding of elevated Feno, and especially the presence of nasal polyposis (Fig 3) make him an excellent candidate for corticosteroid-sparing dupilumab. Dupilumab has the additional advantage of impressive efficacy in chronic rhinosinusitis and nasal polyposis, with a bigger impact on patient-reported outcome measures than mepolizumab or omalizumab,^78^ the two other biologics with a label for chronic rhinosinusitis with nasal polyposis.^79^^,^^80^ Our choice of biologic therefore would be dupilumab, recognizing that a small risk of symptomatic hypereosinophilia exists.^81^

### Case Report 4 Variation

This patient is a 55-year-old man treated with mepolizumab since 2020 because of a history of severe eosinophilic asthma. The prebiologic blood eosinophil count was 0.63 × 10^9^/L. Initial response was very good, with progressive loss of efficacy in the past year after the easing of COVID-19 social distancing. The last asthma attack, occurring while the patient was taking mepolizumab, was assessed in the clinic: he demonstrated airflow limitation with a 750-mL (25%) decrement in FEV_1_ compared with the usual values (3 L → 2.25 L; 65% predicted value). Blood eosinophil count was 0.10 × 10^9^/L and Feno was 112 ppb (historically always raised 60-160 ppb). Sputum culture findings were negative and an adherence check showed continued adherence to optimal inhaled therapy. A sputum differential count showed persistent eosinophilic airway inflammation with a relative eosinophil count of 16% (normal, < 2%-3%).

#### Case Report 4 Discussion

This case report is typical of the clinical conundrum of the modern-day asthmatologist: when to switch a patient from one biologic to another, to which biologic, and why. Unfortunately, to our knowledge, no study has assessed a switching protocol among the six approved biologics. However, an increasing body of real-world experience in this area supports decision-making.^50^^,^^82^^,^^83^ After at least 6 months of initiating a biologic and troubleshooting for issues with the injection itself (ie, failure to refrigerate or to inject it correctly), the following three lines of evidence can assist in our understanding of biologic-refractory asthma attacks.

First, it is important to recognize that a proportion of people receiving biologic therapy show decreasing adherence to ICS treatment. Considering that mepolizumab-emergent nonadherence to ICS predicts poor clinical response,^84^ it is essential to check adherence before switching,^85^ as was done in this case. Although benralizumab may suppress very high Feno levels,^86^ in the SHAMAL trial, a worrisome decline in FEV_1_ was noted in people who decreased the ICS dose to as-needed budesonide-formoterol, and this was associated temporally with an increase in Feno.^34^ No significant decrease in FEV_1_ was noted in people receiving low-to-moderate dose ICS therapy. For these reasons, we ensure that patients receiving biologics take at least a moderate dose of ICS.

Second, mepolizumab-emergent asthma attacks are heterogenous events that may have persistent eosinophilic airway inflammation. In the prospective observational mepolizumab exacerbations study,^87^ 45 people experienced an initial exacerbation with mepolizumab assessed multimodally, including a sputum differential cell count: one-half showed eosinophilia when using a cutpoint of sputum eosinophil count of ≥ 2% (high sputum eosinophil count). The high sputum eosinophil count group showed greater airflow limitation, a slightly higher blood eosinophil count than expected with mepolizumab (median, high sputum eosinophil count vs low sputum eosinophil count: 0.07 × 10^9^/L vs 0.03 × 10^9^/L), and overtly raised Feno (median, 54 ppb vs 24 ppb). In fact, an Feno measurement of < 20 ppb showed a 100% positive predictive value for predicting noneosinophilic, infectious events, whereas greater Feno predicted sputum eosinophilia at exacerbation (≥ 50 ppb such as in this patient: 77% positive predictive value to identify high sputum eosinophil count events). Interestingly, oral prednisolone has extensive additional biologic and antiinflammatory effects at the stable state in patients taking mepolizumab,^88^ perhaps supporting its use in high-Feno mepolizumab-emergent asthma attacks. Whether low-Feno attacks in the setting of anti-IL-5 therapy benefit from prednisolone is under investigation.^89^

Recruitment of eosinophils to the airways may persist, despite near-total systemic depletion of mepolizumab. It is possible that even a few mepolizumab-refractory eosinophils show increased sensitivity to alarmin stimulation via suppressor of cytokine signaling 3 upregulation,^90^, ^91^, ^92^ that bone marrow depletion by mepolizumab is incomplete, or that other cytokines such as IL-13 play a role in eosinophil recruitment. It is unknown if this relates to the development of antimepolizumab antibodies. However, data show that marked improvement may occur in patients after a mepolizumab to benralizumab switch, suggesting that complete eosinophil depletion is necessary in certain patients.^83^

Third, discontinuation of mepolizumab reverts the clinical picture back to what it was before biologics. Indeed, trial patients with severe eosinophilic asthma who discontinued the drug experienced significant increases in blood eosinophils, symptoms, and number of exacerbations within 3 to 6 months after the last dose of mepolizumab.^93^ It is likely that upstream-acting biologics like tezepelumab also have temporary effects, based on the 9-month discontinuation data of the Extension Study to Evaluate the Safety and Tolerability of Tezepelumab in Adults and Adolescents With Severe, Uncontrolled Asthma (DESTINATION).^94^ Hence, it is always worth checking that the exacerbating patient has continued biologic injections.

#### Case Report 4 Conclusions

This patient with severe eosinophilic asthma demonstrated an initial good response to mepolizumab, followed by loss of biologic efficacy despite continued adherence to ICS therapy. Mepolizumab-emergent asthma attacks showed persistent type 2 airway inflammation (with eosinophilia and high Feno). A switch of biologics is recommended. In the absence of comparative data, no clear choice exists between striving for an agent with an Feno-lowering feature (dupilumab or tezepelumab) targeting the predominant epithelial profile of this patient^75^ or striving for greater tissular and systemic eosinophil depletion (benralizumab),^83^ although this choice is less likely to be effective given the increase in Feno highlighting more of an IL-13-mediated pathway. Thus, a trial of either dupilumab or tezepelumab should be considered for this patient, and if no response results, then switching to the alternate would be reasonable.

## Conclusions

The development of multiple biologics in asthma has led to major benefits for patients and new therapeutic dilemmas for asthmatologists. Certainly, confirming the diagnosis of asthma, conducting an adherence check, and completing the workup with objective measures of the type 2 immune response is necessary in all patients. Nevertheless, as made evident by this case-based discussion, in the presence of overlapping prescribing criteria and in the absence of head-to-head comparisons, multiple choices often are reasonable to consider, with certain biologics more suited to address certain comorbidities and biomarker profiles than others. Indeed, the blood eosinophil count and, when available, Feno, are important aspects of the decision process for patients with purely severe asthma and no other treatable comorbidity. These considerations, and the newer problem of when, why, and how to switch biologics, are important research questions that need to be addressed. It is also hoped that other therapies targeting alternate pathways in severe asthma are developed that may offer further hope to these patients.

## Funding/Support

Supported by the Association Pulmonaire du Québec’s Research Chair in Respiratory Medicine and the 10.13039/501100020951Fonds de Recherche du Québec - Santé.

## Financial/Nonfinancial Disclosures

The authors have reported to *CHEST* the following: S. C. reports nonrestricted research grants from the NIHR Oxford BRC, the Quebec Respiratory Health Research Network, the Fondation Québécoise en Santé Respiratoire, AstraZeneca, bioMérieux, and Sanofi-Genyme-Regeneron; support as the Association Pulmonaire du Québec’s Research Chair in Respiratory Medicine and as a clinical research scholar of the Fonds de Recherche du Québec; speaker honoraria from AstraZeneca, GlaxoSmithKline, Sanofi-Regeneron, and Valeo Pharma; consultancy fees from FirstThought, AstraZeneca, GlaxoSmithKline, Sanofi-Regeneron, Access Biotechnology, and Access Industries; and sponsorship to attend or speak at international scientific meetings by or for AstraZeneca and Sanofi-Regeneron. He is an advisory board member and will have stock options for Biometry, Inc., a company that is developing an Feno device (myBiometry). He advised the Institut National d’Excellence en Santé et Services Sociaux for an update of the asthma general practice information booklet for general practitioners. D. J. J. has received advisory board and speaker’s fees from AstraZeneca, Boehringer Ingelheim, Novartis, Teva, GSK, Sanofi/Regeneron, and Chiesi outside of the submitted work. I. D. P. has received speaker’s honoraria for speaking at sponsored meetings from AstraZeneca, Boehringer Ingelheim, Aerocrine AB, Almirall, Novartis, Teva, Chiesi, Sanofi/Regeneron, Menarini, and GSK in the last 5 years and has received payments for organizing educational events from AstraZeneca, GSK, Sanofi/Regeneron, and Teva. He has received honoraria for attending advisory panels with Genentech, Sanofi/Regeneron, AstraZeneca, Boehringer Ingelheim, GSK, Novartis, Teva, Merck, Circassia, Chiesi, and Knopp and has received payments to support Food and Drug Administration approval meetings from GSK. He has received sponsorship to attend international scientific meetings from Boehringer Ingelheim, GSK, AstraZeneca, Teva, and Chiesi. He has received a grant from Chiesi to support a phase 2 clinical trial in Oxford. He is copatent holder of the rights to the Leicester Cough Questionnaire and has received payments for its use in clinical trials from Merck, Bayer, and Insmed. In 2014-2015 he was an expert witness for a patent dispute involving AstraZeneca and Teva. M. E. W. reports grants and personal fees from Novartis, Sanofi, GSK, and Cohero Health; personal fees from Regeneron, Genentech, Sentien, Restorbio, Equillium, and Genzyme; grants, personal fees, and nonfinancial support from Teva; personal fees and nonfinancial support from Boehringer Ingelheim; and grants, personal fees, and nonfinancial support from AstraZeneca.
